# Draft Genome Sequence of *Streptomyces* sp. Strain JV178, a Producer of Clifednamide-Type Polycyclic Tetramate Macrolactams

**DOI:** 10.1128/genomeA.01401-17

**Published:** 2018-01-04

**Authors:** Yunci Qi, John M. D’Alessandro, Joshua A. V. Blodgett

**Affiliations:** a Department of Biology, Washington University in St. Louis, St. Louis, Missouri, USA

## Abstract

Here, we report the draft genome sequence of *Streptomyces* sp. JV178, a strain originating from Connecticut (USA) garden soil. This strain produces the polycyclic tetramate macrolactam compounds clifednamides A and B. The draft genome contains 10.65 Mb, 9,045 predicted protein coding sequences, and several natural product biosynthetic loci.

## GENOME ANNOUNCEMENT

Members of the *Streptomyces* genus of Gram-positive filamentous bacteria produce diverse secondary metabolites, including two-thirds of clinical antibiotics (1). *Streptomyces* genomes have been shown to contain a rich repertoire of small-molecule-encoding biosynthetic loci (2). A high proportion of sequenced *Streptomyces* strains encode the biosynthesis of polycyclic tetramate macrolactams (PTMs) (3). *Streptomyces* sp. strain JV178 was isolated from a Connecticut (USA) garden soil sample during a screen to discover new PTM compounds (4). We have sequenced its genome to further understand clifednamide-type PTM biogenesis.

Strain JV178 was grown in Trypticase soy broth supplemented with 0.6% glycine at 28°C. Genomic DNA was extracted by phenol-chloroform extraction as described elsewhere (5). Sequencing was performed on a MiSeq platform (2 × 301-bp reads [Illumina, Inc., San Diego, CA, USA]) on a paired-end library prepared using a high-throughput library preparation kit (Kapa Biosystems). Adapter sequences and low-quality reads (quality score < 0.05) were removed using CLC Genomics Workbench (CLC Bio-Qiagen, Aarhus, Denmark). The trimmed reads totaled 3,064 Mb, corresponding to approximately 287-fold coverage. *De novo* genome assembly was performed in CLC Genomics Workbench, and resulting contigs having less than 200 bp or 3× average coverage were discarded. The resulting draft genome contains 10,650,097 bp in 759 contigs (*N*_50_, 540,451 bp), with a G+C content of 71.0%. Gene prediction and annotation by the Rapid Annotations using Subsystems Technology version 2.0 pipeline (6, 7) predicted 9,045 protein coding sequences and 126 RNA genes.

Automated secondary metabolism analysis using antiSMASH version 4.0 (8) and PRISM version 3.0 (9) predicted 39 biosynthetic gene clusters. Six of these matched known clusters for concanamycin (10), coronafacic acid (11), marineosins (12), melanin, ectoine, and anticapsin (13). Using this sequence, the clifednamide biosynthetic cluster was also found (14), along with clusters predicted to encode five terpenes, three polyketides, three nonribosomal peptides, eight hybrid polyketide/nonribosomal peptides, four siderophores, five lantipeptides, a single lasso peptide, and three bacteriocins. Multilocus sequence alignment using *atpD*, *gyrB*, *recA*, *rpoB*, and *trpB* concatenates (15) identified the closest sequenced relative of strain JV178 as *Streptomyces torulosus* strain NRRL B-3889, consistent with prior 16s rRNA gene analysis (3). These results place this strain in the *S. scabiei* clade (16), which contains several plant-associated *Streptomyces* species, including the potato pathogen *S. scabiei* 87.22 (17). While we failed to detect homologs of known pathogenicity loci, such as *txtAB* (18), eight genes were predicted to encode the plant-growth hormone auxin. The absence of recognizable pathogenicity loci and the predicted ability to produce the plant modulators concanamycin (10), coronafacic acid (11), and auxin suggest a plant-associated but nonpathogenic lifestyle. These observations suggest potential roles for the clifednamides, whose targets are still unknown.

### Accession number(s).

The draft genome sequence of *Streptomyces* sp. strain JV178 was deposited in DDBJ/ENA/GenBank under the accession number PEKU00000000. The version described in this paper is the first version, PEKU01000000.
